# Severe tracheal and bronchial collapse in adults with type II mucopolysaccharidosis

**DOI:** 10.1186/s13023-016-0425-z

**Published:** 2016-04-26

**Authors:** M. Rutten, P. Ciet, R. van den Biggelaar, E. Oussoren, J. G. Langendonk, A. T. van der Ploeg, M. Langeveld

**Affiliations:** Department of Pulmonology, Erasmus University Medical Center, Rotterdam, The Netherlands; Department of Radiology, Erasmus University Medical Center, Rotterdam, The Netherlands; Department of Paediatric Pulmonology, Sophia Children’s Hospital, Erasmus University Medical Center, Rotterdam, The Netherlands; Center for Lysosomal and Metabolic Diseases, Erasmus University Medical Center, Rotterdam, The Netherlands; Division of Pharmacology, Vascular and Metabolic Diseases, Department of Internal Medicine, Erasmus University Medical Center, Rotterdam, The Netherlands

**Keywords:** Adults, Airway obstruction, Mucopolysaccharidoses, Mucopolysaccharidosis type II, Respiratory function tests, Respiratory system abnormalities/complications, Tomography, X-Ray computed, Trachea abnormalities

## Abstract

**Background:**

Mucopolysaccharidosis type II (MPSII) patients frequently suffer from dyspnoea caused by restrictive airway disease due to skeletal abnormalities as well as glycosaminoglycans (GAG) accumulation at different levels of the airway, including the trachea. In this study we describe the extent of the tracheal and bronchial narrowing, the changes in airway diameter during respiration and the effects of these obstructions on respiratory function in adult MPSII patients.

**Methods:**

Five adult MPSII patients (mean age 40 years) were included. Pulmonary function tests and in- and expiratory chest CT scans were obtained. Cross-sectional areas of trachea and main bronchi were measured at end-inspiration and -expiration and percentage collapse was calculated.

**Results:**

There was diffuse narrowing of the entire intra-thoracic trachea and main bronchi and severe expiratory collapse of the trachea in all patients. At 1 cm above the aortic arch the median % collapse of the trachea was 68 (range 60 to 77 %), at the level of the aortic arch 64 (range 21–93 %), for the main bronchi this was 58 (range 26–66 %) on the left and 44 (range 9–76 %) on the right side. The pulmonary function tests showed that this airway collapse results in obstructive airway disease in all patients, which was severe (forced expiratory volume <50 % of predicted) in four out of five patients.

**Conclusion:**

In adult MPS II patients, central airways diameters are strikingly reduced and upon expiration there is extensive collapse of the trachea and main bronchi. This central airways obstruction explains the severe respiratory symptoms in MPSII patients.

**Electronic supplementary material:**

The online version of this article (doi:10.1186/s13023-016-0425-z) contains supplementary material, which is available to authorized users.

## Background

Mucopolysaccharidosis type II (MPSII or Hunter syndrome; OMIM +309900) is a rare, X-linked disease characterized by lysosomal accumulation of the glycosaminoglycans (GAGs) heparan and dermatan sulfate due to deficiency of the enzyme iduronate-2-sulfatase. The intralysosomal accumulation of GAGs causes cellular dysfunction resulting in progressive damage to various organs and tissues. In approximately 75 % of patients, progressive neurological and cognitive decline is the most disabling manifestation of the disease and these severely affected patients die in first or second decade of life. The remaining patients, further referred to as non-neuronopathic patients, have normal cognitive function [1, 2]. Other prominent signs and symptoms of MPSII are typical facial features, Ear-Nose-Throat (ENT) infections and hearing defects, cardiac valve pathology, respiratory disease, skeletal changes, reduced joint mobility and hernias. Extent of organ involvement and disease progression are highly variable between patients [3].

Dyspnea is a frequent symptom in MPSII patients of multifactorial etiology. First, GAGs accumulate in mucosa and soft tissues of the throat leading to enlargement of the larynx, tonsils, adenoids and tongue resulting in reduced upper airway mobility and obstruction. Second, the altered thorax shape and limited mobility of the ribs and intercostal soft tissue often causes restrictive airway disease [4]. Third, tracheobronchomalacia causes airway obstruction. Tracheobronchial narrowing has been previously described in children and adults with MPSII [5–9].

To date changes in airway diameter during respiration in MPSII patients has not been systematically studied. After observing a high prevalence of severe respiratory symptoms in adult MPSII patients, we decided to investigate the tracheobronchial collapsibility of these patients by performing pulmonary function tests (PFTs) and in-and expiratory chest CT scans.

## Methods

In this study, all adult MPSII patients attending the adult metabolic outpatient clinic at the Erasmus MC, Rotterdam, the Netherlands were included. All patients signed informed consent for a long-term follow-up study, of which pulmonary evaluation is a part. This study was approved by the Medical Ethical Committee of the Erasmus University Medical Center. Written informed consent was obtained from all participants.

### Pulmonary function tests

PFTs were obtained in all patients as part of routine follow-up. Spirometry was performed in accordance with American Thoracic Society standards [10]. Values of percent-predicted for spirometry were calculated using reference values based on age, height, sex and race [11].

The basic parameters used to properly interpret lung function were the vital capacity (VC), forced expiratory volume in 1s (FEV1), FEV1/VC ratio and total lung capacity (TLC) [11]. The carbon monoxide diffusing capacity (DLCO) and adjusted DLCO for the measured lung volume (DLCO/VA) assisted to diagnose the underlying disease, when interpreted in conjunction with the lung volumes assessment. Airway obstruction was defined by reduction of the FEV1/VC ratio (also known as Tiffeneau- index) below 70 %. A Tiffenau-index below 70 % depicts a disproportionate reduction of maximal airflow from the lung in relation to the maximal expiratory volume (VC), implying airway narrowing during exhalation. The severity of lung function impairment was based on the forced expiratory volume in 1s as a percentage of the normal predicted value, corrected for sex, age, height and race (FEV1%pred). A restrictive ventilatory defect is characterized by a reduction in total lung capacity (TLC) below 80 % of the predicted value. TLC was measured by Helium [He] dilution (TLC-He) in all patients and additionally by whole-body plethysmography (TLC-Pleth) in a single patient. In body plethysmography, breathing manoeuvres are performed in an airtight box of known volume with fixed barometric pressure. The two techniques for measuring TLC yield similar results for those with normal lung mechanical function but can yield differential results in patients with airflow obstruction. Whole-body plethysmography is more accurate because of the detection of poorly or non-ventilated lung areas, also known as trapped air. A higher TLC measured by bodyplethysmography compared to that measured by Helium dilution is therefore indicative of air trapping [12].

### Airway imaging and imaging analysis

We performed in- and expiratory chest CT scans in all patients. Chest CT was done on different multi-detectors CT scanners, with detectors ranging from 64 (SOMATON Definition AS+, SIEMENS Healthcare, Forchheim, Germany) to 192 (SOMATOM Force, SIEMENS Healthcare, Forchheim, Germany) detectors. CT protocol included an end-inspiratory and -expiratory scan starting from 2 cm above the lung apices to the bases. Instructions for voluntary breath holds were given before scanning by the CT technician. Scanning parameters were: collimation 0.625 mm, 50 % overlap, slice thickness 1 mm, pitch 1, 110 kV tube voltage and tube current adapted to recommended volumetric computer tomography dose index (CTDI_vol_), which is 1.6 mGy per adult subject. For expiratory CTs, image quality was considered sufficient using a tube current fixed at 25 mA with an effective tube current-time product of 10 mAs; producing a lower radiation dose than the inspiratory protocol. No contrast agent was administered. Images were reconstructed with a 2 mm slice thickness as multiplanar reformats (MPR) in the coronal and sagittal planes. Sharp reconstruction kernel filter without under- or overshoot at edges were used for central airway measurements.

**Fig. 1:**
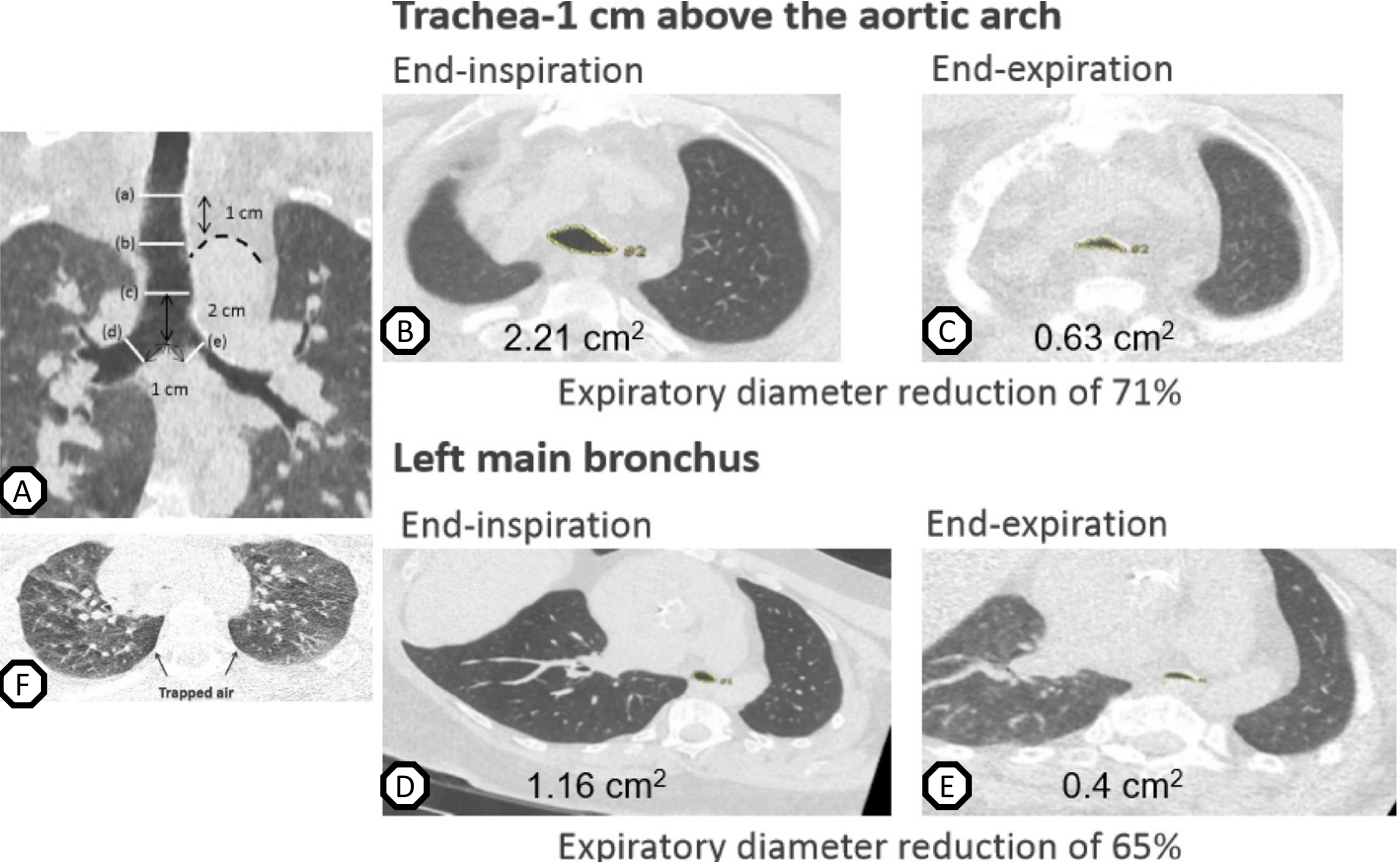
In- and expiratory chest CT scan. **a** Central airways measurements were performed at five locations: (a) 1 cm above aortic arch, (b) aortic arch, (c) 2 cm above carina bifurcation, (d) right main bronchus, (e) left main bronchus. **b** Tracheal cross section at “b” (1 cm above the aortic arch) in patient 2. Note abnormal “bell-shaped” trachea in the end-inspiration image. **c** At end expiration there is almost complete collapse of the trachea (same patient). **d** Bronchial cross section at “e”(left main bronchus) at end inspiration (patient 2) and **e** at end expiration (same patient). **f** Presence of trapped air at expiration (patient 1)

Images were transferred to the Myrian dicom viewer (Intrasense, Montpellier, France). Cross-sectional area (CSA) was measured using a manual tracing tool perpendicular to the tracheal midline. To obtain images perpendicular to the midline, double-oblique reformats were obtained by the axial MPR with sharp kernel (i.e. B50f, I70f, B75f) [13] (Ciet et al. 2015). Measurements of CSA were obtained at five levels throughout the central airways: 1 cm above the aortic arch, at the level of the aortic arch, 2 cm above the carina, 1 cm below carina in the main left and right bronchi (Fig. 1a). CSA measurements were performed with constant lung window (WL -600; WW 1200) both in the end-inspiratory and at end-expiratory images by a single radiologist with extensive experience in thoracic radiology. The percentage airway collapse was calculated according the formula:

$$ \Delta\ \%=\frac{\mathrm{CSAins}-\mathrm{CSAexp}}{\mathrm{CSAins}}\ast 100. $$

Where Δ % is the percentage collapse, and CSAins and CSAexp are the CSA in the end-inspiratory and expiratory images respectively.

## Results

### Patient characteristics

Five adult MPSII patients were enrolled in the study (mean age 40 years, age range 29–50). All patients were male and had normal cognitive function. Four out of five patients were diagnosed during childhood (age range 3 to 9 years), one patient was diagnosed at adult age (age 45 years). In the latter patient the disease was the least severe and only at age 45 the cluster of ENT problems, recurrent pulmonary infections, cardiac valve pathology and skeletal problems were recognized as being caused by MPS II. In all five patients, enzyme replacement therapy (ERT) was started and all but one received ERT at the time of the pulmonary evaluation. Duration of ERT at time of evaluation ranged from 3 months to 7 years. The single untreated patient discontinued ERT 4 years prior to the evaluation because of recurrent anaphylactic reactions during enzyme infusion. At the time, he had been treated for 2 years. Patient characteristics are summarized in Table 1. Three patients received nightly ventilatory support, in one this was bilevel positive airway pressure (BiPAP) and in the other two continuous positive airway pressure (CPAP) (Table 2).

**Table 1:** Patient characteristics

	Patient 1	Patient 2	Patient 3	Patient 4	Patient 5
Mutation	c.806A > T	c.1024 C > T	c.182 C > A	c.1265G > A	c.1122C > T
Age (years)	49	45	43	31	29
Age at diagnosis	7	45	7	9	3
Height (m)	1.71	1.76	1.61	1.52	1.44
Weight (kg)	91	111	60	51	44
Age start ERT therapy	47	45	37	25	25
Currently on ERT therapy	Yes	Yes	No	Yes	Yes
Duration of ERT therapy (years)	2	0.25	2	7	4

*N/A* not applicable, *ERT* enzyme replacement therapy

**Table 2:** Pulmonary function tests

	Patient 1	Patient 2	Patient 3	Patient 4	Patient 5
VC max (L)	4.01	3.79	1.83	1.5	2.01
VC max (%)	91	78	46	40	61
FEV1 (L)	1.32	2.33	1.36	0.55	1.18
FEV1 (%)	38	62	43	18	41
Tiffeneau (FEV1/VC) (%)	33	61	74	37	59
TLC-He (L)	5.52	5.38	2.84	3.6	2.94
TLC-He (%)	84	77	49	71	66
TLC-Pleth			3.50		
TLC-Pleth (%)			60		
DLCO (%)	73	94	46	44	43
DLCO/VA(%)	108	140	132	109	129
Age at time of start ventilatory support	50	N/A	40	31	N/A
Type of ventilatory support	CPAP	N/A	BiPAP	CPAP	N/A

*VC* vital capacity (in litre and as % of predicted), *FEV1* forced expiratory volume in 1 s (in litre and as % of predicted), *TLC-He* total lung capacity measured by helium dilution (in litre and as % of predicted), *TLC-Pleth* total lung capacity measured by bodyplethysmography (in liter and as % of predicted), *DLCO* carbon monoxide diffusing capacity (% of predicted), *DLCO/VA* carbon monoxide diffusing capacity adjusted for lung volume (% of predicted). *CPAP* continuous positive airway pressure, *BiPAP* bilevel positive airway pressure

### Pulmonary function

Table 2 shows the results of the PFT measurements, performed at a time point closest to the airway imaging date (median time gap 14 days). In patients 1, 2, 4 and 5 the lung function analysis showed airway obstruction (FEV1/VC <0.7). In three patients this was accompanied by restriction as the total lung capacity (TLC) measured was below 80 % of predicted. Thus, mixed obstructive and restrictive airway disease was present in these patients. Patient 1 showed only obstructive airway disease. Patient 3 appeared to have only restrictive airway disease but the marked difference between TLC measured by body plethysmography (60 %) and TLC-He (49 %) was suggestive of trapped air, which is usually seen in the context of airway obstruction. The concave shape of the expiratory part of the flow volume loop was indeed indicative of airflow obstruction (Additional file 1: Figure S1). Thus, patient three also had obstructive airway disease.

In patient 4 the airway impairment was very severe (FEV1%pred <35). In patients 1, 3 and 5 the airway impairment was severe (FEV1%pred <50). Patient 2 had moderate airway impairment (FEV1%pred 61). This was the patient diagnosed at adulthood, with an overall mild MPSII phenotype.

Diffusing capacity was normal in all patients. The low DLCO but normal DLCO/VA excludes parenchymal disease and indicates extraparenchymal abnormalities. In MPSII these extraparenchymal abnormalities are the anomalous thorax shape and mechanics caused by thoracic spine and rib deformities [4].

### Airway imaging

All patients successfully completed the CT protocol. In the inspiratory imaging, all patients had diffusely narrowed and thickened trachea and main bronchi. The trachea showed an abnormal triangular shape with flattening of the cartilaginous portion and enlargement of the pars membranacea (Fig. 1b). As a result, the CSA was already significantly reduced in the end-inspiratory images.

Severe expiratory collapse of the trachea was seen in all patients. Table 3 described the central airways measurements at five different locations at end-inspiration and -expiration and the percentage collapse for each patient. Median percentage collapse at different levels of the airway were: 68 % at 1 cm above the aortic arch), 64 % at the level of the aortic arch, 58 % 2 cm above the carina. Main bronchi collapse measured at 1 cm below carina was 58 % for the left main bronchus and 44 % for the right main bronchus (Fig. 1b-e). Because of the already diffusely narrowed central airway lumen in inspiration, the remaining airway lumen at end expiration is much smaller than if the collapse would have occurred in a normally shaped trachea. In the most severely affected patient, there was an almost complete closure of the trachea during expiration. The expiratory chest scan also showed the presence of trapped air in all but one patient (Fig. 1f).

**Table 3:** In- and expiratory cross sectional areas at different levels of the central airway

Location	Patient 1	Patient 2	Patient 3	Patient 4	Patient 5
1 cm above aortic arch	0.83 → 0.32 (61 %)	2.21 → 0.63 (71 %)	1.66 → 0.67 (60 %)	0.81 → 0.26 (68 %)	0.84 → 0.19 (77 %)
Aortic arch	1.02 → 0.39 (62 %)	2.08 → 0.75 (64 %)	1.58 → 0.11 (93 %)	0.35 → 0.12 (66 %)	0.87 → 0.69 (21 %)
2 cm above carina	1.04 → 0.53 (49 %)	2.07 → 0.98 (53 %)	1.59 → 0.27 (83 %)	0.3 → 0.3 (0 %)	0.80 → 0.53 (34 %)
1 cm below carina left main bronchus	0.54 → 0.4 (26 %)	1.16 → 0.4 (66 %)	1.04 → 0.41 (61 %)	0.31 → 0.13 (58 %)	0.53 → 0.34 (36 %)
1 cm below carina right main bronchus	0.52 → 0.29 (44 %)	1.33 → 0.65 (51 %)	1.55 → 0.37 (76 %)	0.54 → 0.49 (9 %)	0.64 → 0.56 (13 %)

Before arrow: cross sectional area in cm at end inspiration, after the arrow: cross sectional area at end expiration in cm, in brackets percentage airway lumen collapse between end-inspiratory and end-expiratory imaging

## Discussion

This study shows extensive collapse of the trachea and main bronchi, resulting life threatening airway obstruction in adult MPSII patients. Airway collapse was present in all patients included in this study. Adult MPSII patients have severely reduced central airway calibre caused by diffuse tracheobronchomalacia. Previous studies in younger MPSII patients [14, 15] (Wooten et al. 2013 median age 9 years *n* = 30, Lin et al. 2014 mean age 18 years *n* = 12) reported PFT outcomes. In these younger patients, mainly restrictive airway disease was observed. In the study of Lin et al. [15], the oldest MPSII patients (age 24 and 33 years) also showed mixed restrictive and obstructive airway disease. In that study, there was a clear negative correlation between age and FEV1, indicative of worsening of pulmonary function with increasing age. Our study shows that in non-neuronopathic MPSII patients aged 30 years or older, pulmonary function is more severely compromised than in younger patients and that this is mainly due to progression of the obstructive component of the airway disease. The cause of this progression is thought to be due to the increasing tracheal and bronchial obstruction and collapse.

The observed severe expiratory collapse of the trachea and main bronchi can explain the high rate of deaths caused by airway impairment in adult MPSII patients [2]. In one of our patients (patient 3), there was almost complete closure of the trachea upon expiration (Table 3). The clinically most severely affected patients (patient 3 and 4) were the patients with by far the smallest expiratory diameter of the trachea at the level of the aortic arch and 2 cm above the carina (Table 3).

In patient 3, nightly bilevel positive airway pressure (BiPAP) was started when respiratory insufficiency was detected (mean transcutaneous CO_2_ 8.5 KPa) resulting in improvement of symptoms and normalisation of the pCO_2_ levels. In patient 4 there were progressive respiratory symptoms despite early antibiotic treatment in case of suspected pulmonary infection, oral steroid therapy to reduce airway inflammation and inhaled bronchodilator therapy. Because of progressive airway obstruction due to severe tracheobronchial collapse together with increased sputum production due to reduced mucocilliary clearance, nightly CPAP therapy was started with a significant reduction in work of breathing and improvement in respiratory symptoms. In this patient, placement of a tracheostomy or tracheal stent was discussed, but not pursued because of the severity and extensiveness of the airway obstruction, involving the entire trachea and main bronchi.

There is an additional value of airway imaging compared to PFTs alone in the assessment of respiratory symptoms in MPSII patients. For example, in patient 3 standard lung function tests could not explain his severe respiratory symptoms. Additional body plethysmography suggested obstructive airway disease and trapped air that was confirmed by CT scanning. Thus, the combination of these tests was necessary to correctly identify the origin of the severe respiratory symptoms.

In addition to management of the respiratory symptoms, knowledge of airway form and function in MPSII patients is important for planning surgical procedures. Pre-anesthesia airway evaluation should include fibroscopic inspection of the upper airway and PFTs. Based on this study we also recommend to perform (dynamic) airway imaging by chest CT scan to assess airway morphology. In this manner a comprehensive multidisciplinary approach to peri-operative airway management can be achieved. Possible concerns related to radiation exposure deriving by the use of CT is likely overcome by the severity of the central airway obstruction. Moreover, thanks to magnetic resonance imaging (MRI), this limitation might be resolved, as shown in a recent study in pediatric patients with tracheobronchomalacia [16].

Respiratory pathology in MPS is thought to arise from the accumulation of GAGs at different levels of the airway. Histopathological studies in MPS II have shown GAG accumulation in adenoidal and supraglottic tissues [17], but accumulation in tracheal tissues has, to our knowledge, only been shown in MPS I and mucolipidosis patients [18]. Collapsibility of the trachea has not been studied in other forms of MPS then MPS II. Gross histopathological examination of tracheas of MPSII patients showed flattening of the tracheal cartilage with loss of the anterioposterior diameter [5]. Whereas in most sites involved in MPSII GAG accumulation results in stiffness and reduced mobility (e.g. cardiac valves, joints, larynx, skin) in the trachea the result is softening and weakness of the supporting cartilage, resulting in airway collapse. A factor that might be responsible for this loss of structural integrity of the cartilage could be inflammation. In a rodent model for MPS VI, in which full collapse of the trachea usually occurs by 9 months of age, two forms of anti-inflammatory treatment resulted in an increased tracheal lumen and a thicker tracheal wall compared to untreated rats [19, 20]. As such, anti-inflammatory therapy may be of value in the treatment of the severe tracheabronchomalacia, since ERT does not seem to prevent the occurrence of this life-threatening disease manifestation.

## Conclusions

In adult MPSII patients, severe tracheal and bronchial collapse result in obstructive airway disease leading to trapped air. In combination with the restrictive lung disease present in these patients, this can lead to respiratory insufficiency and complete respiratory arrest, especially during airway infections. Because of the very specific nature of the airway pathology tailored therapy, such as modified non-invasive ventilation, is required.

## Additional file

Additional file 1: Figure S1. Flow-volume curve patient 3. In this patient, the FEV1/VC was above 70 %, which does not indicate obstructive airway disease. However, the concave shape of the expiratory part of the flow-volume loop is compatible with airway obstruction which fits with the imaging results. The discrepancy between the FEV1/VC value and the other findings is explained by the presence of trapped air. (PPTX 53 kb)

## Abbreviations

BiPAP: bilevel positive airway pressure
CPAP: continuous positive airway pressure
CSA: cross-sectional area
CTDI_vol_: volumetric computer tomography dose index
DLCO: carbon monoxide diffusing capacity
DLCO/VA: adjusted DLCO for the measured lung volume
ENT: ear-nose-throat
ERT: enzyme replacement therapy
FEV1: forced expiratory volume in one second
FEV1%pred: forced expiratory volume in one second as a percentage of the normal predicted value, corrected for sex, age, height and race
GAGs: glycosaminoglycans
He: helium
MPR: multiplanar reformats
MPSII: mucopolysaccharidosis type II
PFTs: pulmonary function tests
TLC: total lung capacity
TLC-He: TLC measured by helium dilution
TLC-Pleth: TLC measured by whole-body plethysmography
VC: vital capacity
