# Supplementary figures and images for: Severe tracheal and bronchial collapse in adults with type II mucopolysaccharidosis
Source: Orphanet J Rare Dis. 2016 Apr 26;11:50. doi: 10.1186/s13023-016-0425-z (PMC4845328; doi:10.1186/s13023-016-0425-z)

## Slide 1
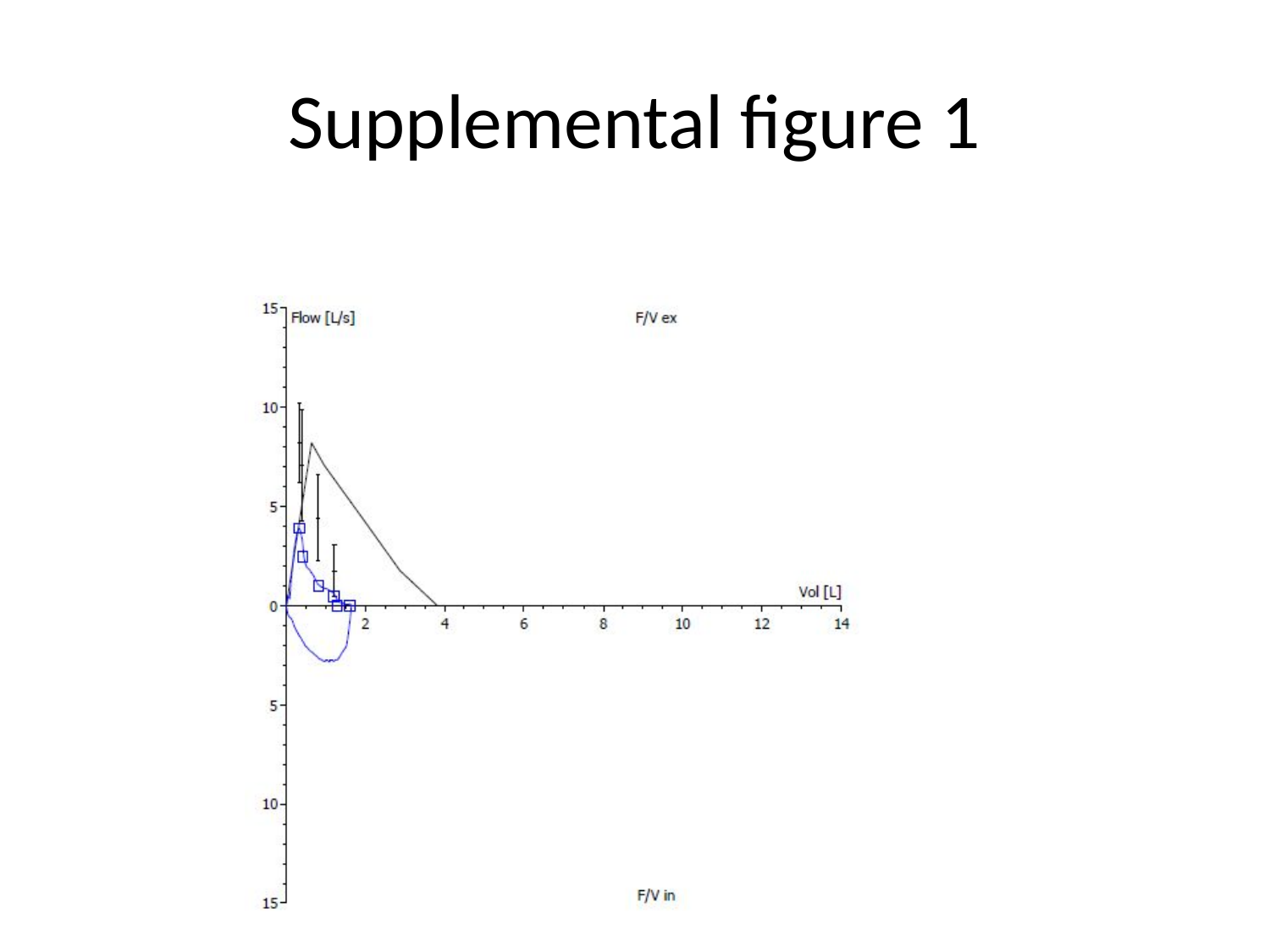

# Supplemental figure 1

Supplement: Additional file 1: Figure S1. — Flow-volume curve patient 3. In this patient, the FEV1/VC was above 70 %, which does not indicate obstructive airway disease. However, the concave shape of the expiratory part of the flow-volume loop is compatible with airway obstruction which fits with the imaging results. The discrepancy between the FEV1/VC value and the other findings is explained by the presence of trapped air. (PPTX 53 kb) [file 13023_2016_425_MOESM1_ESM.pptx]
